# Identification and taxonomic characterization of *Bordetella pseudohinzii* sp. nov. isolated from laboratory-raised mice

**DOI:** 10.1099/ijsem.0.001540

**Published:** 2016-12-01

**Authors:** Yury V. Ivanov, Bodo Linz, Karen B. Register, Jeffrey D. Newman, Dawn L. Taylor, Kenneth R. Boschert, Soazig Le Guyon, Emily F. Wilson, Lauren M. Brinkac, Ravi Sanka, Suellen C. Greco, Paula M. Klender, Liliana Losada, Eric T. Harvill

**Affiliations:** ^1^​ Department of Veterinary and Biomedical Sciences, Pennsylvania State University, University Park, PA, USA; ^2^​ Center for Vaccines and Immunology, University of Georgia, Athens, GA, USA; ^3^​ United States Department of Agriculture, Agricultural Research Service, National Animal Disease Center, Ames, IA, USA; ^4^​ Department of Biology, Lycoming College, Williamsport, PA, USA; ^5^​ Division of Comparative Medicine, Washington University, St. Louis, MO, USA; ^6^​ Lee Kong Chian School of Medicine and Singapore Centre on Environmental Life Sciences Engineering, Nanyang Technological University, Singapore, Singapore; ^7^​ J. Craig Venter Institute, Rockville, MD, USA

**Keywords:** *Bordetella pseudohinzii*, *B. hinzii*, novel species, pathogen

## Abstract

*Bordetella hinzii* is known to cause respiratory disease in poultry and has been associated with a variety of infections in immunocompromised humans. In addition, there are several reports of *B. hinzii* infections in laboratory-raised mice. Here we sequenced and analysed the complete genome sequences of multiple *B. hinzii*-like isolates, obtained from vendor-supplied C57BL/6 mice in animal research facilities on different continents, and we determined their taxonomic relationship to other *Bordetella* species. The whole-genome based and 16S rRNA gene based phylogenies each identified two separate clades in *B. hinzii*, one was composed of strains isolated from poultry, humans and a rabbit whereas the other clade was restricted to isolates from mice. Distinctly different estimated DNA–DNA hybridization values, average nucleotide identity scores, gene content, metabolic profiles and host specificity all provide compelling evidence for delineation of the two species, *B. hinzii* – from poultry, humans and rabbit – and *Bordetella pseudohinzii* sp. nov. type strain 8-296-03^T^ (=NRRL B-59942^T^=NCTC 13808^T^) that infect mice.

*Bordetella* species have historically been subdivided into the ‘classical’ bordetellae represented by the respiratory pathogens *Bordetella bronchiseptica*, *Bordetella pertussis*, *Bordetella parapertussis* and six less extensively studied species (Goodnow, 1980; Mattoo & Cherry, 2005; Diavatopoulos *et al.*, 2005). The latter, ‘non-classical’ bordetellae, include *Bordetella hinzii* (Vandamme *et al.*, 1995), *Bordetella holmesii* (Weyant *et al.*, 1995), *Bordetella petrii* (von Wintzingerode *et al.*, 2001), *Bordetella avium* (Kersters *et al.*, 1984), *Bordetella trematum* (Vandamme *et al.*, 1996) and ‘*Bordetella ansorpii*’ (Ko *et al.*, 2005), in addition to the recently proposed species *Bordetella sputigena*, *Bordetella bronchialis* and *Bordetella flabilis* from human (Vandamme *et al.*, 2015) and *Bordetella muralis*, *Bordetella tumbae* and *Bordetella tumulicola* from environmental samples (Tazato *et al.*, 2015). Unlike the classical bordetellae, which infect and cause disease of the respiratory tract of their natural hosts, the non-classical species are associated with a wide range of disease presentations. For example, while *B. holmesii* and *B. avium* cause respiratory disease, *B. trematum* and ‘*B. ansorpii*’ have been isolated from wound infection. *B. hinzii* is known to cause respiratory disease in poultry (Vandamme *et al.*, 1995; Register & Kunkle, 2009) and has been associated with infections in immunocompromised humans, including bacteremia (Cookson *et al.*, 1994), septicemia (Kattar *et al.*, 2000), respiratory disease (Spilker *et al.*, 2008; Funke *et al.*, 1996) and chronic cholangitis (Arvand *et al.*, 2004).

In addition to infecting poultry and humans, there are several reports of *B. hinzii* infections in laboratory-raised mice. One report describes a spontaneous infection of specific-pathogen-free mice (C57BL/6) with *Bordetella* sp. (Garhart, 2002) that was later identified as *B. hinzii* (Garhart, 2002). Isolation of an organism identified as *B. hinzii* from a laboratory mouse with bronchopneumonia in Japan (Hayashimoto *et al.*, 2008) led to an assessment of this organism’s prevalence in multiple Japanese animal facilities that identified 195 isolates from 44 different facilities (Hayashimoto *et al.*, 2012). Further studies reported isolation of *B. hinzii*-like organisms from a mouse that was imported from Australia to an animal research facility in Germany (Benga *et al.*, 2014), from lungs of a mouse in the USA (Spilker *et al.*, 2014) and from a mouse at an animal facility in Malaysia (Loong *et al.*, 2016). In addition to laboratory mice, *B. hinzii*-like bacteria were also found in a wild Tanezumi rat, the most common rodent in Southeast Asia (Jiyipong *et al.*, 2013). Oropharyngeal isolates from vendor-supplied C57BL/6 mice at a different animal facility in the USA also tested positive for an organism initially identified as *B. hinzii* but revealed evidence that it might be distinct from this species in important ways (Ivanov *et al.*, 2015).

We sequenced the genomes of *B. hinzii* type strain LMG 13501^T^ and of three strains isolated from mice. Each sample was sequenced to at least 75-fold coverage on an Illumina MiSeq, the reads were assembled using Newbler 2.8 or SPAdes v 3.1.1 and the resulting number of contigs per isolate ranged from 48 to 93. We reconstructed a whole-genome phylogeny as previously described (Park *et al.*, 2012), processing the genomes into sets of overlapping 54 bp sequences that were subsequently mapped onto the reference genome of *B. bronchiseptica* strain RB50 (complete genome accession BX470250.1), using SSAHA2 v. 2.5.4 (Ning, 2001). The resulting alignments were analysed with the maximum-likelihood algorithm implemented in RaxML v. 7.0.4. (Stamatakis, 2006) with the general time-reversible model for nucleotide substitution with gamma-distributed rates of heterogeneity (GTRGAMMA), and the final tree was visualized with FigTree v. 1.4 (http://tree.bio.ed.ac.uk/software/figtree/).

The whole-genome phylogeny is based on all *Bordetella* species that have currently been sequenced, namely the classical bordetellae *B. bronchiseptica*, *B. parapertussis* and *B. pertussis* and the more recently described, non-classical species *B. hinzii, B. holmesii*, *B. avium*, *B. trematum* and *B. petrii* (Tables 1 and S1, available in the online Supplementary Material). While the closely related classical bordetellae form a tight clade, the genome sequences of other classified species cluster in distinctly separate branches of the tree and appear to be monophyletic with limited intra-species sequence diversity (Fig. 1a). However, genomes of *B. hinzii* separate into two clades, one consisting of a single strain each isolated from mice at Case Western Reserve University in Cleveland, OH, USA (strain CWR-1), at Washington University in St. Louis, MO, USA (strain 8-296-03^T^) or at the Heinrich Heine University in Dusseldorf, Germany (strain 228/11). The other clade contains nine strains isolated either from poultry (strains OH87 BAL007II, CA90 BAL1384 and 4161 and the *B. hinzii* type strain LMG 13501^T^), from immunocompromised humans (strains 1277, L60, F582 and H568) or from a rabbit (strain 5132; Table 1).

**Fig. 1.**
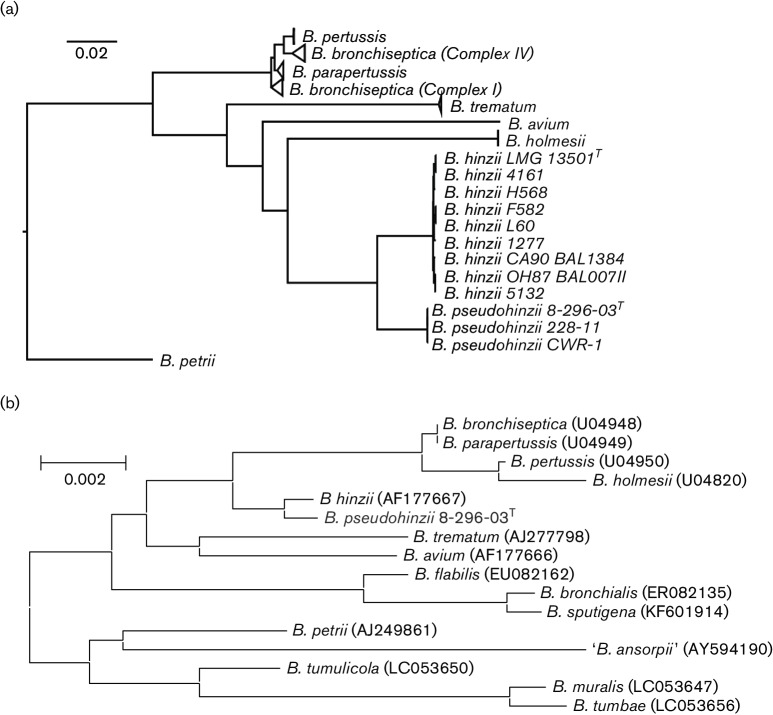
Phylogenetic structure (neighbour-joining trees) according to (a) a genome-wide sequence alignment and (b) 16S rRNA gene sequences of *Bordetella* species. The (GenBank/EMBL/DDBJ/PIR) accession numbers for the Whole Genome Shotgun sequences of *B. hinzii* strain LMG 13501^T^, *Bordetella pseudohinzii* strain 8-296-03^T^, *Bordetella pseudohinzii* strain CWR-1 and *Bordetella pseudohinzii* strain 228/11 are LRUJ00000000, JHEP00000000, LRSQ00000000 and LRSP00000000, respectively. Accession numbers for genomes of all other species and strains are listed in Table S1.

**Table 1.** Description of isolates and their genome information

Species and strain	Host	Year of isolation	Country of isolation	Repository ID	Genome, GenBank ID	Reference
*B. pseudohinzii* 8-296-03^T^	Mouse	2008	USA	NRRL B-59942^T^ NCTC 13808^T^	JHEP00000000.2	Ivanov *et al.* (2015)
*B. pseudohinzii* CWR-1	Mouse	2002	USA	na	LRSQ00000000.1	Garhart (2002)
*B. pseudohinzii* 228/11	Mouse	2011	Germany*	na	LRSP00000000.1	Benga *et al.* (2014)
*B. hinzii* LMG 13501^T^	Chicken	1995	Australia	ATCC 51783^T^	LRUJ00000000.1	Vandamme *et al.* (1995)
*B. hinzii* 4161	Turkey	1979 or prior	USA	NRRL B-59941	JHER00000000.1	Register & Kunkle (2009)
*B. hinzii* CA90 BAL1384	Turkey	1990	USA	NRRL B-59939	JHEO00000000.1	Register *et al.* (2015)
*B. hinzii* OH87BAL007II	Turkey/chicken	Unknown	USA	NRRL B-59935	JHEM00000000.1	Register *et al.* (2015)
*B. hinzii* 1277	Human	1992	Switzerland	NRRL B-59938	JHES00000000.1	Funke *et al.* (1996)
*B. hinzii* L60	Human	1994 or prior	USA	NRRL B-59936	JHEN00000000.1	Cookson *et al.* (1994)
*B. hinzii* F582	Human	1994	USA	na	CP012076.1	Weigand *et al.* (2015)
*B. hinzii* H568	Human	2010	USA	na	CP012077.1	Weigand *et al.* (2015)
*B. hinzii* 5132	Rabbit	1990	Hungary	NRRL B-59940	JHEQ00000000.1	Register & Kunkle (2009)

*Isolate 228/11 was recovered from a mouse that was imported to Germany from Australia.

Within-clade pairwise comparisons revealed 9495±3004 SNPs among the nine *B. hinzii* genomes with average nucleotide identity (ANI) scores of 99.53±1.28 % which were estimated using the ANI calculator (Goris *et al.*, 2007). In contrast, the three strains isolated from mice are genetically extremely monomorphic as their genomes contain only 31±10 pairwise SNPs that result in an ANI of 100±0.11 %. Across clades, genomes differ by 116 464±265 pairwise SNPs and have an ANI of 92.89±2.86 %.

To further assess the degree of delineation between the two groups of genomes, we estimated DNA–DNA hybridization (DDH) using the Genome-to-Genome Distance calculator, a tool that infers genome-to-genome distances between pairs of genomes using a blast-based approach (Meier-Kolthoff *et al.*, 2013). Within-group DDH estimates are very high with 100±0.00 % among the three genomes obtained from mouse isolates and 97.5±1.65 % among the nine genomes from other sources. In contrast, between-group DDH against representative genomes of the two groups was estimated at 52.2±0.20 % (Table S2). The between-group estimates of both ANI and DDH are below the accepted thresholds for the delineation of species, 95 % for ANI (Goris *et al.*, 2007) and 70 % for estimated DDH (Wayne *et al.*, 1987), suggesting that the three isolates from mice, which substantially differ from other *B. hinzii* isolates, represent a novel species. Due to its close relationship with *B. hinzii*, we refer to the novel species as *B. pseudohinzii* sp. nov.

In order to further relate *B. pseudohinzii* sp. nov. to other bordetellae, we extracted an internal fragment of the *nrdA* gene, which was previously shown to reliably differentiate named *Bordetella* species (Spilker *et al.*, 2014), and we compared its sequence against the MLST database (pubmlst.org/bordetella/). All three *B. pseudohinzii* isolates carry *nrdA* allele 189 which was previously described for *Bordetella* genogroup 16 and which was found in isolate HI4681 from a mouse in the USA (Spilker *et al.*, 2014) and isolate BH370 from a mouse in Malaysia (Loong *et al.*, 2016). Likewise, *gyrB* gene sequences (GenBank accession no. AB444711) indicate that Japanese isolate 3224 (Hayashimoto *et al.*, 2008) and 195 additional ‘*B. hinzii*’ that were cultured from tracheal swabs from mice in experimental facilities in Japan (Hayashimoto *et al.*, 2012) may similarly prove to be *B. pseudohinzii*. Indeed, all these isolates from mice possess 16S rRNA gene sequences that are 100 % identical with that from *B. pseudohinzii* 8-296-03^T^ which differs from the 16S rRNA gene of *B. hinzii* by two out of 1522 nucleotides (Fig. 1).

Thus, since previous reports related to the prevalence of a *Bordetella* species in mice seem likely to have confused *B. hinzii* with *B. pseudohinzii*, there is currently no compelling evidence for naturally occurring *B. hinzii* infection in mice. [Whether the *B. hinzii* isolate from a wild rat in Laos (Jiyipong *et al.*, 2013) was accurately speciated remains to be determined.] Instead, *B. hinzii* strains originated from poultry, from immunocompromised humans and from a domesticated rabbit (Table 1). To assess whether *B. pseudohinzii* and *B. hinzii* exhibit host specificity, we evaluated colonization of mouse lungs by either *B. hinzii* (total of seven strains) or *B. pseudohinzii* (two strains). The mouse experiments were carried out in strict accordance with the recommendations in the Guide for the Care and Use of Laboratory Animals of the National Institutes of Health, and the protocol was approved by the Institutional Animal Care and Use Committee at The Pennsylvania State University at University Park, PA (#46284 Bordetella–Host Interactions). For inoculation, 4- to 6-week-old C57BL/6J mice were lightly sedated with 5 % isofluorane (IsoFlo, Abbott Laboratories) and inoculated with 7500 c.f.u. bacteria by gently pipetting 25 µl of the inoculum onto their external nares. To quantify bacterial numbers in the lungs, mice were euthanized by CO_2_ inhalation 7 days after infection, and the lungs were excised. Tissues were homogenized in 1 ml PBS, serially diluted and plated on Bordet-Gengou agar, and colonies were counted after incubation at 37 °C for 2 days. While both bacterial species were able to colonize murine lungs at this time point, *B. pseudohinzii* colonized more efficiently with considerably higher bacterial numbers (Fig. 2). Moreover, colonization by *B. hinzii* was host dependent because numbers of recovered bacteria of *B. hinzii* strains isolated from poultry were on average 10-fold lower than for *B. pseudohinzii* while strains originally isolated from humans were recovered at numbers 2–3 orders of magnitude lower than those observed for *B. pseudohinzii*. In addition, strain 5132 – from a rabbit – that in contrast to other *B. hinzii* isolates failed to colonize poultry in a previous study (Register & Kunkle, 2009) did not establish colonization in mice at all (Fig. 2). These data imply that *B. hinzii* and *B. pseudohinzii* may have different natural hosts and thus occupy different ecological niches.

**Fig. 2.**
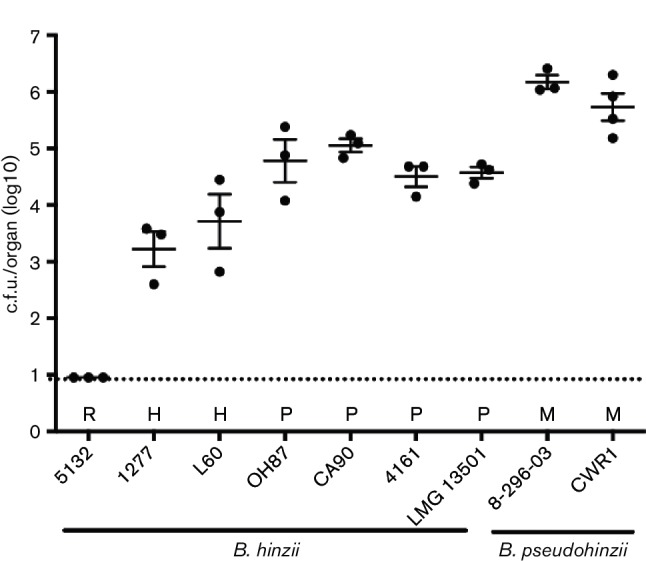
Colonization of murine lungs with either *B. pseudohinzii* or *B. hinzii* 7 days after intranasal inoculation. The dashed line is the limit of detection. The hosts of origin for the *B. hinzii* isolates tested are indicated by H (human), R (rabbit) and P (poultry). *B. pseudohinzii* isolates originated from mouse (M).

Using the narrative method ‘Compute Pangenome’ implemented in KBase, the DOE Systems Biology Knowledgebase (www.kbase.us), we determined clusters of orthologous gene families in *B. hinzii* and *B. pseudohinzii* genomes to analyse presence and absence of genes. The core genome of *B. hinzii* consists of 3776 genes shared among all nine *B. hinzii* genomes. Of those, 3206 genes are also present in the genomes of the three *B. pseudohinzii* isolates (Fig. 3) while 570 are specific to *B. hinzii. B. pseudohinzii* contain 390 genes not present in the genome of any *B. hinzii* strain, including a CRISPR-Cas system that was recently described in *B. pseudohinzii* strain 8-296-03^T^ (Ivanov *et al.*, 2015). We tested additional mouse isolates from the animal facilities at the Case Western Reserve University and Washington University, and all of them contained the CRISPR-Cas system and possessed the two characteristic SNPs in the 16S rRNA gene sequence which differentiate *B. pseudohinzii* from *B. hinzii*.

**Fig. 3.**
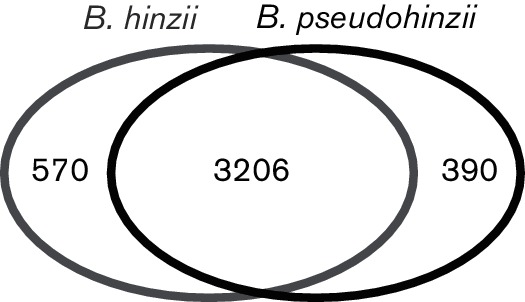
Comparative gene content analysis of *B. pseudohinzii* and *B. hinzii* genomes. Venn diagram compares core genes of nine *B. hinzii* strains (LMG 13501^T^; F582; H568; 1277; L60; OH87 BAL007II; 5132; 4161; CA90 BAL1384) with core genes of three *B. pseudohinzii* strains (8-296-03^T^; 228/11; CWR-1). Numbers correspond to core gene families in common (intersection) or unique-to-species core genes (symmetric difference).

*B. pseudohinzii* strain 8-296-03^T^ and *B. hinzii* strain OH87 BAL007II were grown overnight at 37 °C in Stainer-Scholte broth (Stainer & Scholte, 1970) and stained with 2 % (w/v) of uranyl acetate to take images with a FEI Tecnai Spirit Bio-Twin transmission electron microscope. Both *B. pseudohinzii* and *B. hinzii* appear as rod-shaped coccobacilli with peritrichous, isokont flagella (Fig. S1). In flagella-mediated motility assays that were performed in Stainer-Scholte medium containing 0.4 % (w/v) of agar (Akerley *et al.*, 1992), both species showed swimming motility after 24 h of incubation at 37 °C. While the API 20NE test (bioMérieux) profiled both species as *B. avium* based on the score of 0000067 and thus misidentified them, the GENIII Microbial ID test (Biolog) identified both species as *B. hinzii*. We subjected two strains each (*B. pseudohinzii* strains 8–296-03^T^ and CWR-1 and *B. hinzii* strains LMG 13501^T^ and OH87 BAL007II) to a comprehensive carbon utilization test (PM1 and PM2, Biolog), following the manufacturer's instructions as described previously (Bochner, 2009). Similar to *B. hinzii* (Vandamme *et al.*, 1995) and other bordetellae (Vandamme *et al.*, 1996), *B. pseudohinzii* does not assimilate sugars such as glucose, xylose, fructose, sucrose, lactose, mannose, maltose and galactose. However, this test revealed phenotypic differences distinguishing the two species (Table 2) as only *B. pseudohinzii* utilizes d-tartaric acid (Fig. S2a). In contrast, utilization of d-galactonic acid-γ-lactone was positive for *B. hinzii* isolates and negative for *B. pseudohinzii* (Fig. S2b). Interestingly, *B. hinzii* genomes contain a cluster of five genes that are predicted to encode a d-galactonate transcriptional regulator, a 2-dehydro-3-deoxy-galactonokinase, a 2-dehydro-3-deoxy-6-phosphogalactonate aldolase, a d-galactonate dehydratase and a d-galactonate MFS transporter [the (GenBank/EMBL/DDBJ) locus tag numbers are AXA74_RS01375–AXA74_RS01395], gene products that are presumably involved in the utilization of galactonic acid (Fig. S2c). Presence of these genes in genomes of *B. hinzii* but not *B. pseudohinzii* may explain this metabolic difference.

**Table 2.** Differential biochemical characteristics of the novel species *B. pseudohinzii*

Characteristic	*B. pseudohinzii* 8-296-03^T^	*B. pseudohinzii* CWR-1	*B. hinzii* LMG 13501^T^	*B. avium* ATCC 35086^T^*	*B. bronciseptica* ATCC 193195^T^*
Urease activity	−	−	−	−	+
Oxydase activity	+	+	+	+	+
Haemolysis on sheep blood agar	−	−	−	−	+
Nitrate reduction	−	−	−	−	+
Nitrite reduction	−	−	−	−	+
Assimilation of:					
d-Glucose	−	−	−	−	−
l-Malate	+	+	+	+	−
d-Ribose	+	+	+	−	nd
d-Xylose	+	+	+	−	nd
l-Alanine	+	+	+	−	nd
d-Tartaric acid	+	+	−	−	nd
d-Galactonic acid-γ-lactone	−	−	+	nd	nd
API 20NE profile	0000067	0000067	0000067†	0000067*	1200027*

*Previously published data adopted from Kersters *et al.* (1984) and Vandamme *et al.* (1995).

†According to Vandamme *et al.* (1995), the majority of *B. hinzii* strains have profile 0000077 and only few strains have profile 0000067.

We overlaid bacterial cultures on Bordet-Gengou blood plate antibiotic-containing strips (E-test; bioMérieux) and scored antibiotic resistance after incubation at 37 °C for 24 h (Table S3). In accordance with a previous analysis (Funke *et al.*, 1996), *B. hinzii* strains appeared as a multidrug resistant, showing resistance against four of six tested antibiotics (streptomycin, ampicillin, kanamycin and chloramphenicol), but were susceptible to tetracycline and showed intermediate resistance to gentamicin. The only tested *B. pseudohinzii* strain 8-296-03^T^ showed a similar broad-range resistance (Table S3). In addition, the antimicrobial sensitivity of isolate BH470 from a mouse in Malaysia (Loong *et al.*, 2016), which likely belongs to *B. pseudohinzii* based on the 16S rRNA and *nrdA* gene sequences, was also similar to that observed in *B. hinzii* from human infection (Funke *et al.*, 1996; Fry *et al.*, 2007). Thus, similar to *B. hinzii*, *B. pseudohinzii* as a species may be multidrug resistant, with possible differences between individual strains, suggesting antimicrobial resistance as a trait of the last common ancestor of both species.

Respiratory quinones were analysed by a modification of the procedure described by Reddy *et al.* (2007). The HPLC analysis of extracted lipoquinones showed a single peak with an elution time and absorbance spectrum consistent with ubiquinone 8 (Q8), as seen in other *Bordetella* species (Tazato *et al.*, 2015).

## Description of *Bordetella pseudohinzii* sp. nov.

*Bordetella pseudohinzii* (pseu.do.hin′zi.i Gr. prep. *pseudes* false; N.L. gen. n. *hinzii* of Hinz; N.L. gen. n. *pseudohinzii*, resembling *B. hinzii*). Cells are Gram-stain negative, catalase and oxidase positive and motile bacilli with rounded ends that occur as single units. After 48 h of incubation on Bordet-Gengou agar at 37 °C, colonies are translucent and non-pigmented, with smooth margins, and 1.0–1.5 mm in diameter. Isolates grow in the presence of 4.0 % NaCl, do not show haemolysis on sheep blood agar, utilize d-tartaric acid as a carbon source but do not assimilate d-galactonic acid-γ-lactone. Their genomes contain a transcriptionally active type II-C CRISPR-Cas system that is not present in any other *Bordetella* species sequenced to date. The major respiratory quinone is Q8.

Similar to *B. hinzii*, *B. pseudohinzii* utilizes pyruvate, citrate, α-ketoglutarate, succinate, malate, oxaloacetate, acetate and butyrate. However, another substrate of the TCA cycle, fumarate, appears not to be utilized by either species, presumably due to the lack of the appropriate transporters. Both species further assimilated d-/l-α-glycerol-phosphate, d-arabinose, d-glucosamine, d-ribono-1,4-lactone, d-ribose, d-saccharic acid, d-xylose, l-arabinose, l-lyxose, monomethyl succinate, d-alanine, d-aspartic acid, glycyl-l-aspartic acid, glycyl-l-glutamic acid, glycyl-l-proline, l-alaninamide, l-alanine, l-alanyl-glycine, l-asparagine, l-aspartic acid, l-glutamic acid, l-glutamine, l-leucine, l-ornithine, l-phenylalanine, l-proline, l-serine and l-threonine.

The following substances cannot be utilized: glucose, xylose, fructose, sucrose, lactose, mannose, maltose, galactose, arabitol, cellobiose, dextrin, fucose, galacturonic acid, gluconic acid, glucosaminic acid, mannitol, melezitose, melibiose, psicose, raffinose, sorbitol, tagatose, trehalose, dulcitol, fructose 6-phosphate, gentiobiose, glucose 1-phosphate, glucose 6-phosphate, glycerol, erythritol, inulin, lactitol, lactulose, laminarin, rhamnose, sorbose, maltitol, maltotriose, mannan, 3-methyl glucose, inositol, palatinose, pectin, salicin, sedoheptulosan, stachyose, turanose, xylitol, α-/β-/γ-cyclodextrin, methyl α-d-galactoside, methyl α-d-glucoside, methyl α-d-mannoside, allose, *N*-acetyl-d-galactosamine, *N*-acetyl-d-glucosamine, *N*-acetyl-d-glucosaminitol, *N*-acetyl-β-d-mannosamine, methyl β-d-galactoside, methyl β-d-glucoside, methyl β-d-xyloside, chondroitin sulfate C, 2,3-butanone, 3-hydroxy 2-butanone, acetamide, arbutin, octopamine, d-lactic acid methyl ester, glucuronamide, sec-butylamine, 2-aminoethanol, 2-hydroxy benzoic acid, 4-hydroxy benzoic acid, bromo succinic acid, capric acid, caproic acid, citraconic acid, citramalic acid, d-glucuronic acid, glycolic acid, glyoxylic acid, malonic acid, melibionic acid, mucic acid, *N*-acetyl-neuraminic acid, oxalic acid, propionic acid, quinic acid, sebacic acid, α-keto valeric acid, methyl β-d-glucuronic acid, γ-amino butyric acid, γ-hydroxy butyric acid, δ-amino valeric acid, amygdalin, d-/l-carnitine, d-serine, d-threonine, gelatin, glycine, hydroxy-l-proline, l-arginine, l-histidine, l-homoserine, l-isoleucine, l-lysine, l-methionine, phenylethylamine, putrescine, tyramine, l-valine, 1,2-propanediol, 2,3-butanediol and adonitol.

The type strain, 8-296-03^T^ (=NRRL B-59942^T^=NCTC 13808^T^), was isolated from an oropharyngeal swab of a C57BL/6 laboratory mouse in the USA in 2008. The DNA G+C content of the type strain is 66.6 mol%. Additional strains have been isolated from laboratory mice in USA (strains CWR-1 and HI4681), in Germany (strain 228-11), in Japan (strain 3224) and in Malaysia (strain BH370).

## Supplementary Data

6827 Supplementary File 1
