# Supplementary material for: Identification and taxonomic characterization of Bordetella pseudohinzii sp. nov. isolated from laboratory-raised mice
Source: Int J Syst Evol Microbiol. 2016 Dec 1;66(12):5452–9. doi: 10.1099/ijsem.0.001540 (PMC5244500; doi:10.1099/ijsem.0.001540)
Supplement: Supplementary File 1 [file ijsem-66-5452-s001.pdf]

B. pseudohinzii
strain 8-296-03^T

B. hinzii
strain OH87 BAL007II

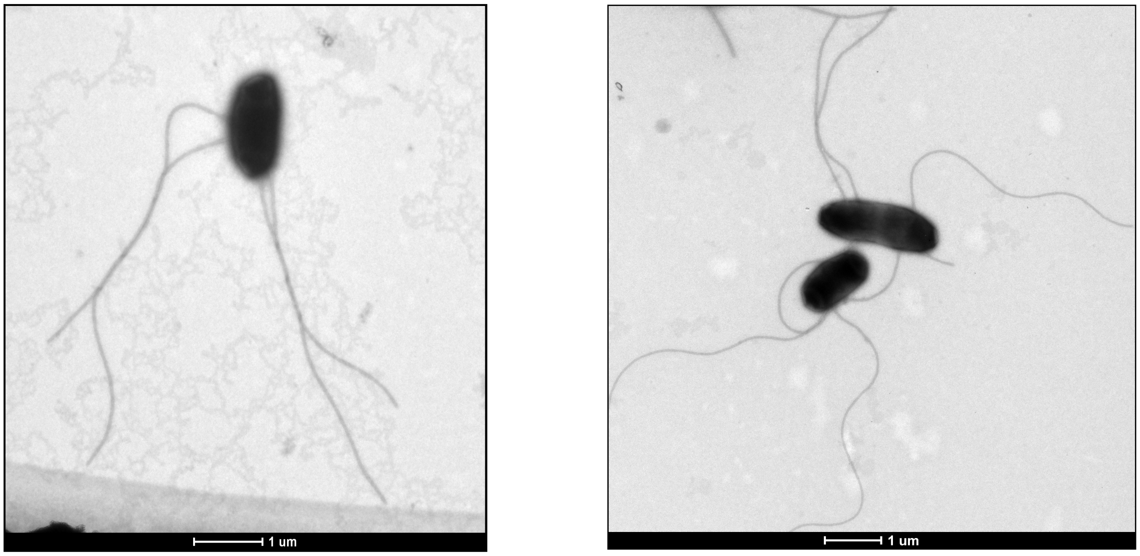

Figure S1. Transmission electron micrographs of *B. pseudohinzii* 8-296-03^T and *B. hinzii* OH87 BAL007II strains. *B. pseudohinzii* and *B. hinzii* are rod-shaped coccobacilli with peritrichous, isokont flagella. The samples were negatively stained with 2% uranyl acetate, the images were taken with a FEI Technai Spirit Bio-Twin transmission electron microscope at 80 KeV at 100,000-fold magnification. Images courtesy of Yury V. Ivanov.

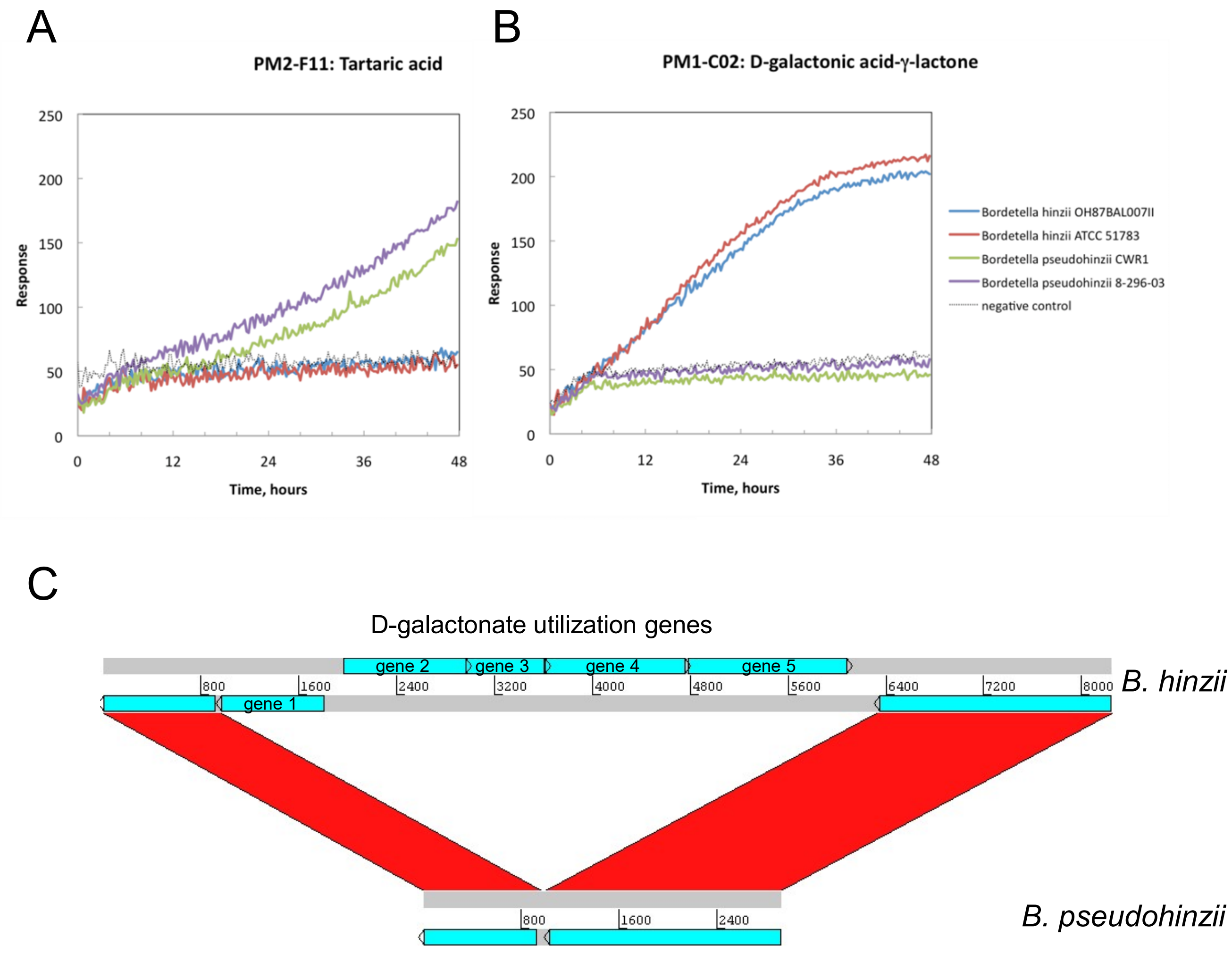

Figure S2. Differential carbon utilization by *B. pseudohinzii* and *B. hinzii*.
A) Assimilation of D-tartaric acid by *B. pseudohinzii*. B) Species-specific utilization of D-galactonic acid-γ-lactone and C) presence of a D-galactonate transcription regulator (gene 1) and a 4-gene operon predicted to encode a 2-dehydro-3-deoxy-galactonokinase (gene 2), a 2-dehydro-3-deoxy-6-phosphogalactonate aldolase (gene 3), a D-galactonate dehydratase (gene 4), and a D-galactonate MFS transporter (gene 5) in *B. hinzii*.

Table S1. Genomes of additional *Bordetella* isolates.

**Species**	**Strain**	**NCBI accession number**	**Reference**
B. bronchiseptica	RB50	BX470250	(Parkhill et al., 2003)
B. bronchiseptica	RB630	JGYA00000000	(Register et al., 2015b)
B. bronchiseptica	3E44	JGWK00000000	(Register et al., 2015b)
B. bronchiseptica	A1-7	JGWO00000000	(Register et al., 2015b)
B. bronchiseptica	B18-5	JGWP00000000	(Register et al., 2015b)
B. bronchiseptica	B20-10725633	JGWQ00000000	(Register et al., 2015b)
B. bronchiseptica	980	JGWM00000000	(Register et al., 2015b)
B. bronchiseptica	CARE970018BB	JGWS00000000	(Register et al., 2015b)
B. bronchiseptica	MBORD849	JGXW00000000	(Register et al., 2015b)
B. bronchiseptica	KM22	JNHR00000000	(Nicholson et al., 2014)
B. bronchiseptica	MBORD595	JGXH00000000	(Register et al., 2015b)
B. bronchiseptica	MBORD785	JGXU00000000	(Register et al., 2015b)
B. bronchiseptica	MBORD839	JGXV00000000	(Register et al., 2015b)
B. bronchiseptica	253	HE965806	(Park et al., 2012)
B. bronchiseptica	MBORD635	JGXK00000000	(Register et al., 2015b)
B. bronchiseptica	MBORD782	JGXT00000000	(Register et al., 2015b)
B. bronchiseptica	MBORD665	JGXL00000000	(Register et al., 2015b)
B. bronchiseptica	MBORD668	JGXM00000000	(Register et al., 2015b)
B. bronchiseptica	MBORD670	JGXN00000000	(Register et al., 2015b)
B. bronchiseptica	MBORD678	JHBQ00000000	(Register et al., 2015b)
B. bronchiseptica	MBORD762	JHBR00000000	(Register et al., 2015b)
B. bronchiseptica	MBORD624	JGXI00000000	(Register et al., 2015b)
B. bronchiseptica	MBORD632	JGXJ00000000	(Register et al., 2015b)
B. bronchiseptica	MBORD731	JGXS00000000	(Register et al., 2015b)
B. bronchiseptica	M435/02/3	JGXE00000000	(Register et al., 2015a)
B. bronchiseptica	M85/00/2	JGXF00000000	(Register et al., 2015a)
B. bronchiseptica	SO10328	JGYB00000000	(Register et al., 2015b)
B. bronchiseptica	1289	CAKS01000000	(Register et al., 2015b)
B. bronchiseptica	MBORD681	JGXP00000000	(Register et al., 2015b)
B. bronchiseptica	MBORD698	JGXQ00000000	(Register et al., 2015b)
B. bronchiseptica	00-P-2796	JGWH00000000	(Register et al., 2015b)
B. bronchiseptica	D756	JGWT00000000	(Register et al., 2015b)
B. bronchiseptica	D989	JGWU00000000	(Register et al., 2015b)
B. bronchiseptica	D993	JGWV00000000	(Register et al., 2015b)
B. bronchiseptica	E010	JGWW00000000	(Register et al., 2015b)
B. bronchiseptica	E012	JGWX00000000	(Register et al., 2015b)
B. bronchiseptica	E013	JGWY00000000	(Register et al., 2015b)
B. bronchiseptica	MBORD591	JGXG00000000	(Register et al., 2015b)
B. bronchiseptica	7E71	JGWL00000000	(Register et al., 2015b)
B. bronchiseptica	CA90 BB02	JHBU00000000	(Register et al., 2015b)
B. bronchiseptica	CA90 BB1334	JGWR00000000	(Register et al., 2015b)
B. bronchiseptica	F-1	JGXA00000000	(Register et al., 2015b)
B. bronchiseptica	F2	JGXB00000000	(Register et al., 2015b)

B. bronchiseptica	MBORD707	JGXR00000000	(Register et al., 2015b)
B. bronchiseptica	MBORD901	JGXX00000000	(Register et al., 2015b)
B. bronchiseptica	OSU054	JHBZ00000000	(Register et al., 2015b)
B. bronchiseptica	OSU095	JGXY00000000	(Register et al., 2015b)
B. bronchiseptica	OSU553	JGXZ00000000	(Register et al., 2015b)
B. bronchiseptica	00-P-2730	JGWG00000000	(Register et al., 2015b)
B. bronchiseptica	E014	JGWZ00000000	(Register et al., 2015b)
B. bronchiseptica	F4563	JGXC00000000	(Register et al., 2015b)
B. bronchiseptica	GA96-01	JGXD00000000	(Register et al., 2015b)
B. bronchiseptica	MBORD675	JGXO00000000	(Register et al., 2015b)
B. bronchiseptica	MO211	JHOJ00000000	(Register et al., 2015b)
B. bronchiseptica	MO275	JHBS00000000	(Register et al., 2015b)
B. bronchiseptica	SBL-F6116	JHBT00000000	(Register et al., 2015b)
B. bronchiseptica	MO149	HE965806	(Park et al., 2012)
B. bronchiseptica	Bbr77	CAKU01000000	(Park et al., 2012)
B. pertussis	Tohama I	BX470248	(Parkhill et al., 2003)
B. pertussis	CS	CP002695	(Zhang et al., 2011)
B. pertussis	STO1-CHLA-0011	AXSP00000000	(Harvill et al., 2013)
B. pertussis	H897	AXSO00000000	(Harvill et al., 2013)
B. pertussis	H918	AXSN00000000	(Harvill et al., 2013)
B. pertussis	H921	AXSM00000000	(Harvill et al., 2013)
B. pertussis	H939	AXSL00000000	(Harvill et al., 2013)
B. pertussis	H973	AXSK00000000	(Harvill et al., 2013)
B. pertussis	STO1-SEAT-0004	AXSJ00000000	(Harvill et al., 2013)
B. pertussis	I002	AXSI00000000	(Harvill et al., 2013)
B. pertussis	I036	AXSH00000000	(Harvill et al., 2013)
B. pertussis	I176	AXSG00000000	(Harvill et al., 2013)
B. pertussis	STO1-CHOC-0008	AXRV00000000	(Harvill et al., 2013)
B. pertussis	STO1-CHOM-0012	AXRU00000000	(Harvill et al., 2013)
B. pertussis	STO1-CNMC-0004	AXSV00000000	(Harvill et al., 2013)
B. pertussis	STO1-CHOC-0016	AXSA00000000	(Harvill et al., 2013)
B. pertussis	STO1-CHOC-0017	AXRZ00000000	(Harvill et al., 2013)
B. pertussis	STO1-CHOC-0018	AXRY00000000	(Harvill et al., 2013)
B. pertussis	STO1-CHOC-0019	AXRX00000000	(Harvill et al., 2013)
B. pertussis	STO1-CHOC-0021	AXRW00000000	(Harvill et al., 2013)
B. pertussis	CHLA-15	AXSD00000000	(Harvill et al., 2013)
B. pertussis	CHLA-13	AXSE00000000	(Harvill et al., 2013)
B. pertussis	CHLA-20	AXSC00000000	(Harvill et al., 2013)
B. pertussis	CHLA-26	AXSB00000000	(Harvill et al., 2013)
B. pertussis	STO1-CHLA-0006	AXSF00000000	(Harvill et al., 2013)
B. pertussis	2250905	AXSU00000000	(Harvill et al., 2013)
B. pertussis	2356847	AXST00000000	(Harvill et al., 2013)
B. pertussis	2371640	AXSS00000000	(Harvill et al., 2013)
B. pertussis	STO1-SEAT-0006	AXSR00000000	(Harvill et al., 2013)
B. pertussis	STO1-SEAT-0007	AXSQ00000000	(Harvill et al., 2013)
B. pertussis	18323	HE965805	(Park et al., 2012)
B. parapertussis	12822	BX470249	(Parkhill et al., 2003)

B. parapertussis	Bpp5	HE965803	(Park et al., 2012)
B. holmesii	H572	JFZY00000000	(Harvill et al., 2014)
B. holmesii	H585	JFZZ00000000	(Harvill et al., 2014)
B. holmesii	H629	JGVZ00000000	(Harvill et al., 2014)
B. holmesii	H635	JGAA00000000	(Harvill et al., 2014)
B. holmesii	H643	JGWD00000000	(Harvill et al., 2014)
B. holmesii	H719	JGWA00000000	(Harvill et al., 2014)
B. holmesii	H785	JGWB00000000	(Harvill et al., 2014)
B. holmesii	H809	JMGZ00000000	(Harvill et al., 2014)
B. holmesii	04P3421	JGWC00000000	(Harvill et al., 2014)
B. holmesii	F627	AOEW01000000	(Tatti et al., 2013)
B. holmesii	H558	AOFR01000000	(Tatti et al., 2013)
B. trematum	CCUG_13902	AWNL01000000	(Shah et al., 2013)
B. avium	197N	AM167904	(Sebaihia et al., 2006)
B. petrii	DSM_12804	AM902716	(Gross et al., 2008)

References

Gross, R., Guzman, C. A., Sebaihia, M., dos Santos, V. A., Pieper, D. H., Koebnik, R., Lechner, M., Bartels, D. & other authors (2008). The missing link: *Bordetella petrii* is endowed with both the metabolic versatility of environmental bacteria and virulence traits of pathogenic Bordetellae. *BMC.Genomics* **9***,* 449.

Harvill, E. T., Goodfield, L. L., Ivanov, Y., Meyer, J. A., Newth, C., Cassiday, P., Tondella, M. L., Liao, P. & other authors (2013). Genome Sequences of 28 *Bordetella pertussis* U.S. Outbreak Strains Dating from 2010 to 2012. *Genome Announc.* **1***,* e01075-13.

Harvill, E. T., Goodfield, L. L., Ivanov, Y., Smallridge, W. E., Meyer, J. A., Cassiday, P. K., Tondella, M. L., Brinkac, L. & other authors (2014). Genome Sequences of Nine *Bordetella holmesii* Strains Isolated in the United States. *Genome Announc.* **2***,* e00438-14.

Nicholson, T. L., Shore, S. M., Bayles, D. O., Register, K. B. & Kingsley, R. A. (2014). Draft Genome Sequence of the *Bordetella bronchiseptica* Swine Isolate KM22. *Genome Announc.* **2***,* e00670-14.

Park, J., Zhang, Y., Buboltz, A. M., Zhang, X., Schuster, S. C., Ahuja, U., Liu, M., Miller, J. F. & other authors (2012). Comparative genomics of the classical *Bordetella* subspecies: the evolution and exchange of virulence-associated diversity amongst closely related pathogens. *BMC Genomics* **13***,* 545.

Parkhill, J., Sebaihia, M., Preston, A., Murphy, L. D., Thomson, N., Harris, D. E., Holden, M. T., Churcher, C. M. & other authors (2003). Comparative analysis of the genome sequences of *Bordetella pertussis*, *Bordetella parapertussis* and *Bordetella bronchiseptica*. *Nat.Genet.* **35***,* 32-40.

Register, K. B., Ivanov, Y. V., Harvill, E. T., Davison, N. & Foster, G. (2015a). Novel, host-restricted genotypes of *Bordetella bronchiseptica* associated with phocine respiratory tract isolates. *Microbiology* **161***,* 580-592.

Register, K. B., Ivanov, Y. V., Jacobs, N., Meyer, J. A., Goodfield, L. L., Muse, S. J., Smallridge, W. E., Brinkac, L. & other authors (2015b). Draft Genome Sequences of 53 Genetically Distinct Isolates of *Bordetella bronchiseptica* Representing 11 Terrestrial and Aquatic Hosts. *Genome Announc.* **3***,* e00152-15.

Sebaihia, M., Preston, A., Maskell, D. J., Kuzmiak, H., Connell, T. D., King, N. D., Orndorff, P. E., Miyamoto, D. M. & other authors (2006). Comparison of the genome sequence of the poultry pathogen *Bordetella avium* with those of *B. bronchiseptica*, *B. pertussis*, and *B. parapertussis* reveals extensive diversity in surface structures associated with host interaction. *J.Bacteriol.* **188***,* 6002-6015.

Shah, N. R., Moksa, M., Novikov, A., Perry, M. B., Hirst, M., Caroff, M. & Fernandez, R. C. (2013). Draft Genome Sequences of *Bordetella hinzii* and *Bordetella trematum*. *Genome Announc.* **1***,* e00838-13.

Tatti, K. M., Loparev, V. N., Ranganathanganakammal, S., Changayil, S., Frace, M., Weil, M. R., Sammons, S., Maccannell, D. & other authors (2013). Draft genome sequences of *Bordetella holmesii* strains from blood (F627) and nasopharynx (H558). *Genome Announc.* **1***,* e00056-13.

Zhang, S., Xu, Y., Zhou, Z., Wang, S., Yang, R., Wang, J. & Wang, L. (2011). Complete genome sequence of *Bordetella pertussis* CS, a Chinese pertussis vaccine strain. *J.Bacteriol.* **193***,* 4017-4018.

Table S2. Pairwise estimated DNA-DNA hybridization values.

		Reference genomes	
		B. pseudohinzii 8-296-03^T	*B. hinzii* LMG13501^T
Query genomes	*B. pseudohinzii*		
	8-296-03^T	100.0	51.9
	CWR-1	100.0	52.0
	228-11	100.0	52.0
	mean ± SD	100.00 ± 0.00	51.97 ± 0.06
	B. hinzii		
	LMG13501^T	51.9	100.0
	4161	52.0	100.0
	CA90_BAL1384	52.5	95.9
	OH87_BAL007II	52.3	95.9
	1277	52.3	97.5
	L60	52.3	97.0
	F582	52.2	97.0
	H568	52.2	98.2
	5132	52.4	95.7
	mean ± SD	52.23 ± 0.19	97.47 ± 1.65

Table S3a. Dilution plating tests for susceptibility of *B. pseudohinzii* sp. nov. and *B. hinzii* to commonly used-in-laboratory antibiotics.

Species	Strain	MIC *, μg ml^{-1}					
		Strep	Amp	Kan	Cam	Tet	Gent
B. pseudohinzii	8-296-03^T	156.3	19.5	19.5	7.8	0.5	9.7
B. hinzii	L60	78.1	19.5	39.1	3.9	0.5	19.5
B. hinzii	5132	39.1	20	20	15.6	1.9	ND

* Abbreviations: MIC, minimum inhibitory concentration; Strep, streptomycin; Amp, ampicillin; Kan, kanamycin; Cam, chloramphenicol; Tet, tetracycline; Gent, gentamycin.

Table S3b. Epsilometer tests for susceptibility of *B. pseudohinzii* sp. nov. and *B. hinzii* to commonly used-in-laboratory antibiotics.

Species	Strain	MIC *, μg ml^{-1}					
		Strep	Amp	Kan	Cam	Tet	Gent
B. psuedohinzii	8-296-03^T	32	14	8	24	0.7	8
B. hinzii	LMG 13501^T	16	24	12	64	0.7	5
B. hinzii	L60	24	12	24	32	2	12
B. hinzii	5132	24	24	20	64	3	5
B. hinzii	OH87 BAL007II	12	24	16	48	2	6
B. hinzii	4161	24	16	32	32	2	8
B. hinzii	1277	24	32	24	32	1.5	12
B. hinzii	CA90 BAL1384	16	32	12	96	3	5

* Abbreviations: MIC, minimum inhibitory concentration; Strep, streptomycin; Amp, ampicillin; Kan, kanamycin; Cam, chloramphenicol; Tet, tetracycline; Gent, gentamycin.
